# Patterns of Sequence and Expression Diversification Associate Members of the *PADRE* Gene Family With Response to Fungal Pathogens

**DOI:** 10.3389/fgene.2020.00491

**Published:** 2020-05-29

**Authors:** Marie Didelon, Mehdi Khafif, Laurence Godiard, Adelin Barbacci, Sylvain Raffaele

**Affiliations:** Université de Toulouse, Laboratoire des Interactions Plantes Micro-organismes (LIPM), Institut National de Recherche pour l’Agriculture, l’Alimentation et l’Environnement (INRAE) – Centre National de la Recherche Scientifique (CNRS), Castanet-Tolosan, France

**Keywords:** plant disease resistance, diversification, DUF4228, intrinsic disorder, pathogenesis-related, gene expression profiling

## Abstract

Pathogen infection triggers extensive reprogramming of the plant transcriptome, including numerous genes the function of which is unknown. Due to their wide taxonomic distribution, genes encoding proteins with Domains of Unknown Function (DUFs) activated upon pathogen challenge likely play important roles in disease. In *Arabidopsis thaliana*, we identified thirteen genes harboring a DUF4228 domain in the top 10% most induced genes after infection by the fungal pathogen *Sclerotinia sclerotiorum.* Based on functional information collected through homology and contextual searches, we propose to refer to this domain as the pathogen and abiotic stress response, cadmium tolerance, disordered region-containing (PADRE) domain. Genome-wide and phylogenetic analyses indicated that PADRE is specific to plants and diversified into 10 subfamilies early in the evolution of Angiosperms. PADRE typically occurs in small single-domain proteins with a bipartite architecture. PADRE N-terminus harbors conserved sequence motifs, while its C-terminus includes an intrinsically disordered region with multiple phosphorylation sites. A pangenomic survey of *PADRE* genes expression upon *S. sclerotiorum* inoculation in *Arabidopsis*, castor bean, and tomato indicated consistent expression across species within phylogenetic groups. Multi-stress expression profiling and co-expression network analyses associated AtPADRE genes with the induction of anthocyanin biosynthesis and responses to chitin and to hypoxia. Our analyses reveal patterns of sequence and expression diversification consistent with the evolution of a role in disease resistance for an uncharacterized family of plant genes. These findings highlight *PADRE* genes as prime candidates for the functional dissection of mechanisms underlying plant disease resistance to fungi.

## Introduction

Wild plants and crops suffer from recurrent attacks by pathogenic microbes, threatening biodiversity and food production. Molecular and genetic studies revealed that plants possess an elaborate immune system able to detect pathogens and activate genetic pathways to mount effective defense responses (Dodds and Rathjen, 2010). Specific defense responses allow plants to cope with microbial pathogens of diverse lifestyles and genotypes that target diverse plant organs (Glazebrook, 2005). In most cases, the activation of plant responses requires extensive transcriptional reprogramming, covering for instance up to 25% of the whole genome in *Arabidopsis thaliana* (Eulgem, 2005). In nature, one of the most frequent forms of plant immunity is designated as quantitative disease resistance (QDR) (Poland et al., 2009; Roux et al., 2014). QDR leads to a full continuum of disease resistance phenotypes in natural plant populations, from very susceptible to largely resistant, and generally involves a large number of genetic loci. Every gene adds a small contribution to form the overall resistance (Roux et al., 2014). Current knowledge of the molecular bases of QDR in plants remains very incomplete, but a few general properties have emerged. First, the molecular functions of QDR genes are very diverse, including for instance transporters (Krattinger et al., 2009), kinases (Derbyshire et al., 2019), proteases (Badet et al., 2017), and genes of unknown function (Fukuoka et al., 2009). Second, the function of QDR genes may not be limited to disease resistance and can include activity in cell morphology (Rajarammohan et al., 2018; Badet et al., 2019), metabolism (Rajarammohan et al., 2018), or embryogenesis (Derbyshire et al., 2019) in certain contexts. Third, QDR responses to a given pathogen species may involve hundreds or even thousands of genes (Corwin et al., 2016; Fordyce et al., 2018). Therefore, pathogen infection triggers extensive reprogramming of the plant transcriptome, including numerous genes the molecular function of which is currently unknown.

Recent progress in high-throughput omics techniques enabled the determination of the sequence of genes and proteins at an unprecedented pace. Homology relationships allow to rapidly transfer functional information from one sequence to another but suffer limitations (Pearson and Sierk, 2005), and our capacity to generate new sequences far exceeds our ability to interpret them. Sequence conservation across large evolutionary distances can identify previously unknown functional domains in proteins, such as in the case of the VASt domain (PF16016) (Khafif et al., 2014, 2017; Gatta et al., 2015). The Protein Family Database (Pfam) gathers protein families by their homology of sequence (El-Gebali et al., 2019). In 2019, the latest Pfam release (32.0) counted 17,929 entries, 3,961 (22%) of them being Domains of Unknown Function (DUFs). DUFs are protein families for which no member has an experimentally characterized function. Systematic structural analyses of DUF proteins revealed that a significant part of DUF proteins likely originate from extreme diversification and neofunctionalization of known protein domains (Jaroszewski et al., 2009). Due to their wide taxonomic distribution and their evolutionary sequence conservation, many DUFs are expected to compose essential proteins (Goodacre et al., 2013). Widely distributed genes encoding proteins with DUFs activated upon pathogen challenge are promising sources of new insight into the evolution and molecular mechanisms of plant disease resistance.

*Sclerotinia sclerotiorum* is a devastating fungal plant pathogen from the Ascomycota division with a necrotrophic lifestyle. It is responsible for the white and stem mold diseases on more than 400 plant species, including crops of high agricultural value like sunflower, soybean, rapeseed, and tomato, among others (Boland and Hall, 1994; Hegedus and Rimmer, 2005). The host range of *S. sclerotiorum* also includes plants from the *Brassicaceae* family, such as the plant model *A. thaliana*. Resistance to *S. sclerotiorum* is typically quantitative with no complete resistance (Perchepied et al., 2010; Mbengue et al., 2016). The molecular bases of QDR to *S. sclerotiorum* are beginning to be elucidated, notably thanks to studies on *A. thaliana*, but remain very patchy (Mbengue et al., 2016). Global gene expression profiling by RNA sequencing revealed 4,703 *A. thaliana* genes significantly induced upon leaf inoculation with *S. sclerotiorum* (Badet et al., 2017), including several genes harboring a DUF4228 domain. Here, we took a survey of DUF4228 homologs across the plant kingdom and identified a few experimental insights into the function of these genes. We propose to refer to this domain as the pathogen and abiotic stress response, cadmium tolerance, disordered region-containing (PADRE) domain to facilitate future reference. We used phylogenetic analyses to document the extent diversity of PADRE sequences and infer scenarios for their evolution. PADRE proteins lack sequence similarity to characterized proteins but harbor a bipartite architecture with conserved motifs in the N-terminal region and a C-terminal region rich in phosphorylated residues and predicted to be intrinsically disordered. Pangenomic expression profiling in thale cress (*A. thaliana*), tomato (*Solanum lycopersicum*), and castor bean (*Ricinus communis*) plants inoculated by *S. sclerotiorum* identified groups of *PADRE* genes that respond to this fungal pathogen in a consistent manner across species. Finally, *AtPADRE* gene expression upon diverse stress treatments and co-expression network reconstruction suggests that several *PADRE* genes could function synergistically in plant defense. Our study reveals that responsiveness to fungal pathogen attack is conserved at the interspecific level in groups of PADRE genes and provides insights into the evolutionary history and functional diversification in this poorly characterized plant gene family.

## Results

### Genes From the DUF4228 Family Are Over-Represented Among Genes Induced Upon *S. sclerotiorum* Inoculation

**FIGURE 1:**
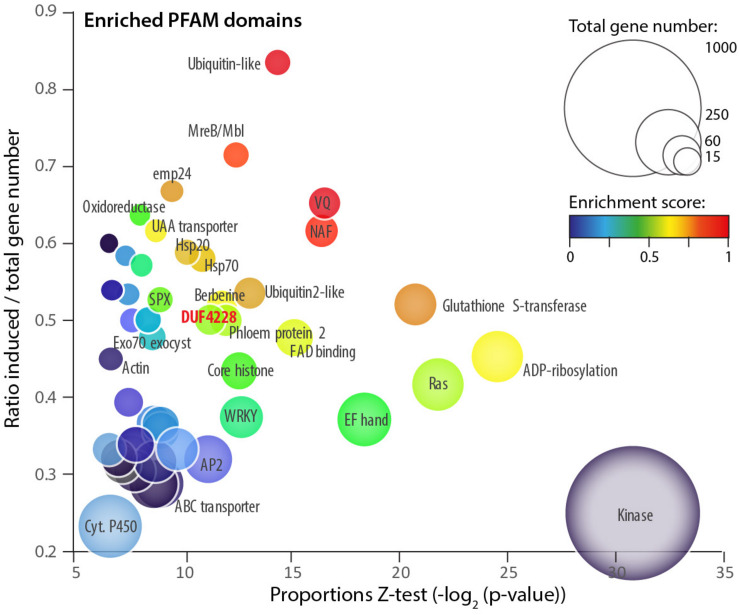
Protein domains enriched among *A. thaliana* genes upregulated upon *S. sclerotiorum* inoculation. Each bubble shows one of 54 PFAM domains significantly enriched (proportion test *p*-value < 0.01) in induced genes (LFC > 1.5, *p*-value < 0.01). Bubbles are sized according to the total number of genes containing the domain in the *A. thaliana* genome. Enrichment is shown as the *p*-value of a proportion *Z*-test for enrichment (*X*-axis), the ratio between the number of induced/total genes (*Y*-axis) and a composite enrichment score (color scale, see the section “Materials and Methods”). The DUF4228 domain is labeled in bold red. Associated raw data corresponds to *Arabidopsis thaliana* samples from GEO accession number GSE106811.

To get insights into plant processes activated during colonization by the fungal pathogen *S. sclerotiorum*, we analyzed RNA-Seq data for *A. thaliana* plants inoculated by *S. sclerotiorum*. Specifically, we focused on protein domains overrepresented among plant genes differentially expressed upon inoculation. To this end, we exploited the RNA sequencing data generated in Badet et al. (2017) (GSE106811). Differential expression analysis identified 4,703 genes significantly induced (log_2_ fold change (LFC) > 1.5, adjusted *p*-value (padj) < 0.01) and 5,812 genes significantly down-regulated (LFC < 1.5, padj < 0.01) in *A. thaliana* during infection by *S. sclerotiorum*. We annotated genes by their protein domains using the Pfam database. Using a proportion *Z*-test (*p*-value < 0.01), we counted 53 protein domains significantly overrepresented among induced genes with at least 10 occurrences in *A. thaliana* genome (Supplementary Table S1 and Figure 1). The ubiquitin-like domain PF14560 showed the strongest enrichment in induced genes (induced/total ratio = 0.83, *p*-val = 4.41e^–05^), and the protein kinase domain PF00069 had the most significant enrichment in induced genes (ratio 0.25, *p*-val 5.02e^–10^). Gene Ontology terms associated with the 74 overrepresented protein domains included defense mechanism and immune response in 64.5% of cases. For instance, 27 out of 72 genes harboring a WRKY domain (PF03106) were induced upon infection by *S. sclerotiorum* (ratio = 0.375, *p*-val 1.43 e^–04^). Other protein domains enriched in induced genes included ubiquitin-like domains (PF10302, PF14560, PF11976, and PF00240), transport-related domains (PF01105, PF08449, and PF03105), calcium binding (PF14658), and heat shock response (PF00011) (Supplementary Table S1).

One domain enriched in induced genes had no known molecular function and was identified as Domain of Unknown Function DUF4228 (ratio 0.5, *p*-val 3.86e^–04^). We identified 28 genes with a DUF4228 domain in the genome of *A. thaliana* (hmmscan e-value < 1E-10, Supplementary Table S2), 19 of them being differentially expressed upon infection by *S. sclerotiorum* (14 induced and 5 down-regulated). The DUF4228 gene AT5G37840 was induced over 1,000 times (LFC 10.22, *p*-val 1.45e^–47^), and 13 genes harboring a DUF4228 domain were in the top 10% most induced genes after infection by *S. sclerotiorum* in *A. thaliana* (LFC > 4.04, Supplementary Table S2). Because of their dramatic induction pattern and although uncharacterized to date, some DUF4228 genes could function in plant defense responses.

### Taxonomic Distribution of the DUF4228/PADRE Domain

**FIGURE 2:**
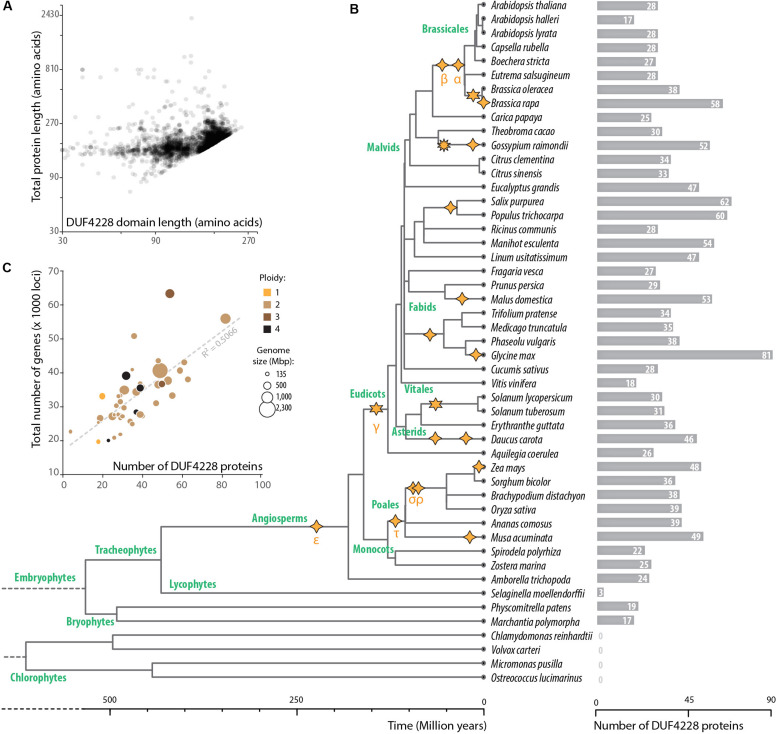
Taxonomic distribution of DUF4228 domains. **(A)** Relationship between total protein length and DUF4228 domain length in 3647 proteins from the Refprot database. **(B)** Timetree and DUF4228 family size for plant species from the Phytozome 12.1 database. The phylogenetic tree and divergence time estimates were obtained using Kumar et al. (2017). Polyploidization events described in the literature (Wang et al., 2019; Xu et al., 2019) are shown as stars with 4 spikes (tetraploidy), 6 spikes (hexaploidy), or 10 spikes (decaploidy). Greek letters correspond to common names attributed to polyploidy events in the literature, starting with events identified in the *Arabidopsis* genome. **(C)** Relationship between DUF4228 family size and total genome size (number of protein-coding loci) in 45 embryophyte species. Bubbles are sized according to genome size in Mbp and colored according to ploidy level.

To document the taxonomic distribution of the DUF4228 domain across the tree of life, we performed a HMM search against the Refprot database of UniProtKB with an alignment of *A. thaliana* DUF4228 proteins as input (Supplementary Datasheet S1). We retrieved 3647 hits distributed in 98 species. As recently reported (Yang et al., 2020), DUF4228 appeared restricted to plants, including mosses, liverworts, and monocot and dicot species. The average size of DUF4228 domains detected in these proteins was 149.7 ± 40.3 amino acids, for proteins of 159.7 ± 37.6 amino acids long (Figure 2A). In good agreement, only 8.4% of proteins harboring a DUF4228 domain were multi-domain proteins. To identify the complete repertoire of DUF4228 in plant proteomes, we performed a HMM search against the Phytozome 12.1 database. Out of the 64 plant proteomes available at the time of our analysis, only the seven Chlorophyte proteomes did not show a single DUF4228 domain, indicating that the emergence of the DUF4228 domain occurred at least 450 million years ago (Figure 2B). Next, we used Timetree to relate the number of DUF4228-containing proteins with time of speciation in 49 plant species. In embryophytes, the number of DUF4228-containing proteins ranged from three (*Selaginella moellendorffii*) to 81 (*Glycine max*) (Figure 2B). A majority of embryophytes (28/45) had between 20 and 40 DUF4228-containing proteins, and there was no striking expansion of DUF4228 in a specific plant lineage. Recent whole genome duplication events were often associated with expanded DUF4228 repertoires, such as in *Brassica rapa*, *Malus domestica*, *G. max*, *Zea mays*, and *Musa acuminata*. Overall, the size of the DUF4228 family was well correlated (*R*^2^ = 0.5066) with the total number of genes per genome across embryophyte species (Figure 2C).

Through homology and keyword searches, we found experimental insights into function for DUF4228 proteins. The *A. thaliana* AT4G37240 protein was identified as interacting with calmodulin proteins CAM4, 6, 7, 8, and 9 (Popescu et al., 2007). In addition, *A. thaliana* AT1G66480 was identified as interacting with Arabidopsis Response Regulator 14 (ARR14) in a yeast two-hybrid screen (Dortay et al., 2008). However, these protein-protein interactions have not been validated by independent approaches. In *Nicotiana tabacum*, the homolog of *A. thaliana AT1G76600* was found responsive to tobacco mosaic virus and wounding and the corresponding protein designated as Pathogenesis-related protein of 23kDa (NtPRp23) (Akiyama et al., 2005). Its ortholog in *N. sylvestris* (LOC104235934) conferred tolerance to cadmium when expressed in yeast (Zhang et al., 2016). Recent work by Yang et al. (2020) revealed that several DUF4228 genes are responsive to drought, cold, or salt abiotic stress. Our analyses reported in this study indicated that several DUF4228 genes are responsive to infection by the fungal pathogen *S. sclerotiorum* and that *A. thaliana* DUF4228 proteins harbor intrinsically disordered regions. Based on this partial functional information and to facilitate further reference, we propose to refer to this family as the pathogen and abiotic stress response, cadmium tolerance, disordered region-containing (PADRE) family.

### Sequence Diversification of the PADRE Domain

**FIGURE 3:**
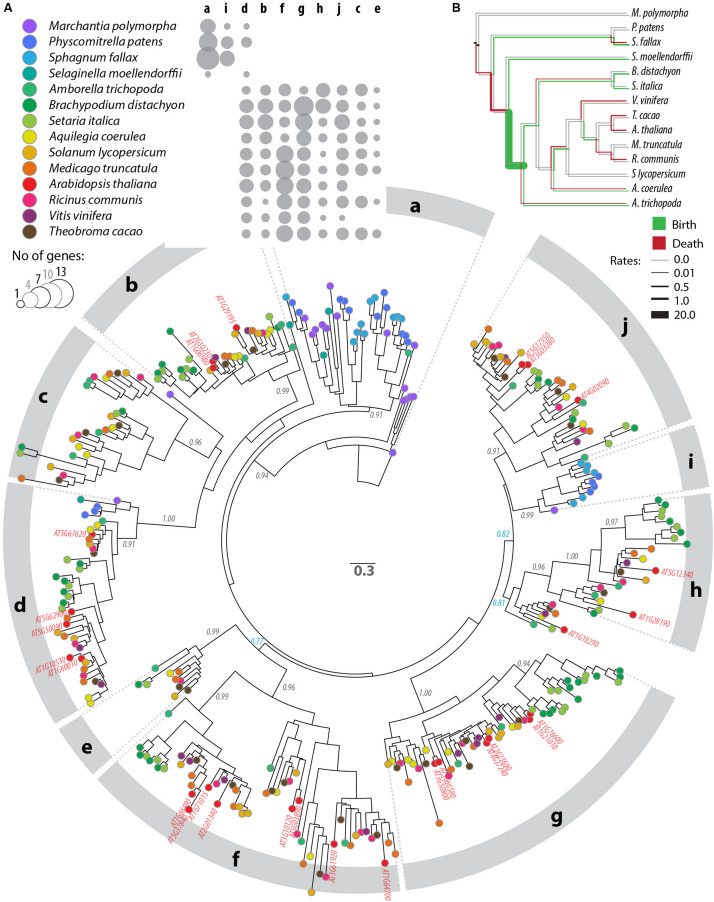
Phylogenetic relationships between DUF4228 proteins in the complete proteome of 13 Embryophyta species. **(A)** Tree obtained by a maximum likelihood analysis, with the number of substitutions per site used as branch length, and branch support determined by an approximate likelihood ratio test (black labels if ≥0.90, blue otherwise). Terminal nodes are color-coded according to plant species (key shown in the upper panel). *A. thaliana* identifiers are labeled in red on the tree. Phylogenetic groups are labeled a to j on the outer circle. The upper panel shows the number of genes per species and per phylogenetic group as bubbles of increasing size. **(B)** Species tree showing rates of PADRE domain gain (green) and loss (red) in the evolution of Embryophyta as calculated with BadiRate (Librado et al., 2012). Neutral branches are shown in gray.

To analyze patterns of sequence diversification among PADRE proteins, we selected 13 plant genomes representative of the major Embryophyta lineages and constructed a phylogenetic tree of PADRE proteins from these species (see the section “Materials and Methods”). For this, we generated a multiple protein alignment including 344 sequences and 116 informative sites located within the PADRE domain (Supplementary Datasheet S2, S3). We used maximum likelihood methods to represent phylogenetic relationships between these 354 PADRE domains as a tree (Figure 3A and Supplementary Datasheet S4). PADRE proteins classified into 10 monophyletic groups (a to j) supported by posterior probabilities ≥ 0.90 and encompassing 10 (clade e) to 55 (clade g) proteins. PADRE sequences diversified strongly since the divergence between Lycophytes and Angiosperms: groups a and i were restricted to Bryophytes and Lycophytes, while groups b, c, e, f, g, h, and j were restricted to Angiosperms. Group d was represented in all species analyzed except *Sphagnum fallax*. Groups b, c, e, f, g, h, and j were represented in all Angiosperm species analyzed, with the exception of groups c and e that were absent from *Arabidopsis thaliana* and *Vitis vinifera*. This suggests that seven PADRE groups existed in the common Angiosperm ancestor and that groups c and e were lost in *A. thaliana* and *V. vinifera*. The number of PADRE groups expanded more rapidly in Angiosperms (reaching 6 and 8 distinct clades per species) than in Bryophytes and Lycophytes (2 or 3 clades per species), indicative of strong diversification of PADRE genes early in the evolution of Angiosperms. To estimate rates of domain birth and death in the PADRE family, we analyzed the species distribution of PADRE phylogenetic group with BadiRate (Librado et al., 2012) (Figure 3B). This revealed two major domain gain events during the emergence of Angiosperms and of core Eudicots and several lineage-specific gain events. Loss events mostly corresponded to the emergence of Tracheophytes and to terminal branches in the Fabids and Malvids clades.

### PADRE Is a Bipartite Domain Including Disordered and Phosphorylated C-Termini

**FIGURE 4:**
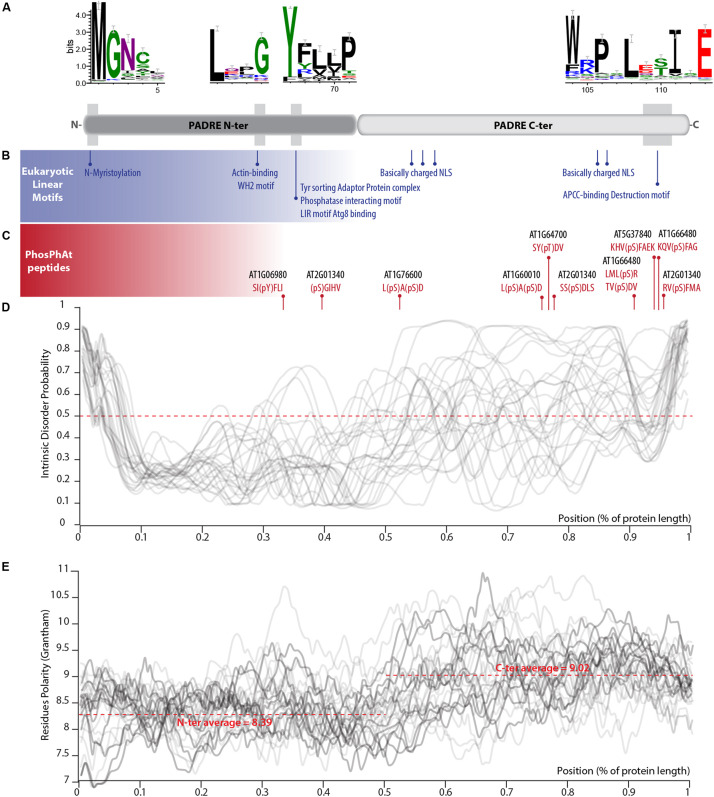
Primary sequence features of PADRE proteins. **(A)** Conserved motifs detected in the alignment of 344 PADRE proteins from 13 Embryophytes. The relative position of these motifs is shown as a light gray shaded area on a diagram representing the PADRE protein structure. **(B)** Eukaryotic linear motifs identified in multiple AtPADRE proteins positioned along the protein diagram shown in **(A)**. **(C)** Phosphorylated residues determined experimentally registered in the PhosPhAt database, positioned along the protein diagram shown in **(A)**. Intrinsic disorder probability **(D)** and Grantham residue polarity **(E)** along the 28 AtPADRE proteins. NLS, nuclear localization signal.

PADRE proteins do not display clear homology to functionally characterized proteins. In our alignment of PADRE proteins from 13 Tracheophyta species, sequence conservation appeared limited to four short motifs of 10 amino acids or less (Figure 4A). These conserved motifs correspond to motifs 1, 3, and 6 identified by Yang et al. (2020). As noted by Yang et al. (2020), additional short sequence motifs were restricted to specific PADRE groups. To get insights into PADRE protein sequence signatures and their potential functional implications, we scanned *A. thaliana* PADRE proteins with the ELM, PhosPhat, PrDOS, and Grantham Polarity calculation tools. First, we used the eukaryotic linear motif (ELM) resource to identify motifs similar to known functional sites in proteins (Gouw et al., 2018) (Figure 4B). Among motifs identified robustly in multiple AtPADRE proteins was an N-myristoylation motif, corresponding to the well-conserved GNXXX motif found at the very N-terminus of PADRE proteins. *In vitro* myristoylation provided experimental for N-myristoylation of AT4G37240 (group G) and AT1G10530 (group D) (Boisson et al., 2003, unpublished result available^1^). Furthermore, AT1G21010 (group G) and ATGG17350 (group J) were identified in plasma-membrane fractions as expected if N-myristoylated (Majeran et al., 2018). The conserved LXXG motif of PADRE proteins overlapped with a WH2 motif for interaction with actin (LIG_Actin_WH2). The conserved YFLLP motif overlapped with a Tyrosine-based signal for interaction with the adaptor protein complex (TRG_ENDOCYTIC_2), a LIR motif for binding to the autophagy protein Atg8 (LIG_LIR_Gen_1), and a protein phosphatase interacting motif (DOC_PP1_RVXF_1). Basic nuclear localization signals were detected at the C-terminus of several PADRE proteins. The C-terminal WRPXLXXIXE motif overlapped with an APCC-binding Destruction motif required for targeting to ubiquitin-mediated proteasome-dependent degradation (DEG_APCC_DBOX_1). ELM also detected numerous putative phosphorylation sites at the C-terminus of PADRE proteins. We took advantage of the PhosPhAt 4.0 database to search for experimentally determined phospho-peptides in PADRE proteins (Durek et al., 2009). We retrieved phospho-peptides from seven AtPADRE proteins from group B (AT1G06980), D (AT1G60010), F (AT1G64700, AT1G66480, AT2G01340, AT5G37840), and G (AT1G76600) (Figure 4C). A large majority of the phosphorylated residues resided in the C-terminal half of the PADRE domain.

We used the PrDOS server (Ishida and Kinoshita, 2007) to predict natively disordered regions in the 28 AtPADRE proteins (Figure 4D). All AtPADRE proteins showed a relatively consistent pattern of disorder probability, indicating a short (∼5 amino acids) N-terminal disordered region followed by an ordered region of ∼60 amino acids and a C-terminal half with high probability of intrinsic disorder. To test whether the structural state of PADRE regions was associated with contrasted amino acid usage at the N and C terminus, we calculated the Grantham Polarity index (Grantham, 1974) along 28 AtPADRE proteins (Figure 4E). In average, the PADRE C-terminal region harbors more polar residues (average index 9.02) than the N-terminal region (average index 8.39).

### Responsiveness to *S. sclerotiorum* Varies Across PADRE Phylogenetic Groups

**FIGURE 5:**
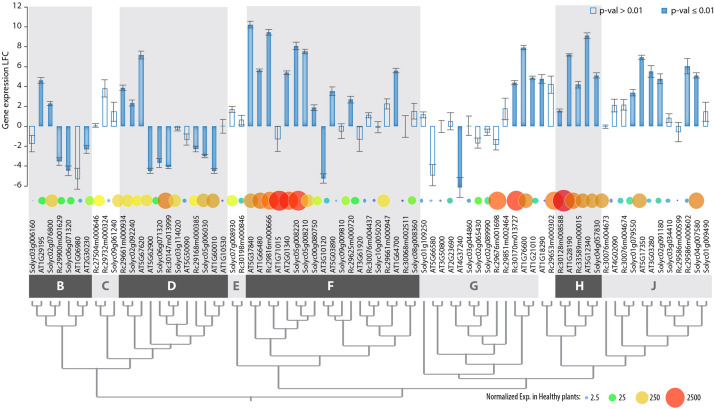
Genome-wide expression of *PADRE* genes in *Arabidopsis thaliana*, *Ricinus communis*, and *Solanum lycopersicum* in response to *S. sclerotiorum* determined by RNA sequencing. Genes are ordered according to phylogenetic relationships shown in Figure 3, with phylogenetic groups delimited by dark and light gray boxes. Bar plot shows LFC of gene expression upon *S. sclerotiorum* inoculation, with error bars showing standard error for LFC over three independent biological experiments. Bars are filled when *p*-value for differential expression determined by DESeq2 analysis is ≤0.01, empty otherwise. Bubbles are sized and colored according to normalized gene expression in healthy plants. Exp., gene expression; LFC, log_2_ fold change of gene expression. The associated raw data is available from GEO accessions GSE106811 and GSE138039.

The clear delineation of phylogenetic groups in the PADRE family and recent investigations of *AtPADRE* gene expression upon abiotic stress (Yang et al., 2020) suggested that *PADRE* genes could have acquired several distinct functions over evolution. Here, we set to investigate whether responsiveness to the fungal pathogen *S. sclerotiorum* contrasts across PADRE phylogenetic groups and whether responsiveness to fungal infection is consistent across plant species. To this end, we analyzed the expression of the *PADRE* gene repertoire of *A. thaliana*, *Solanum lycopersicum*, and *Ricinus communis* by RNA-sequencing in leaves of healthy plants and upon inoculation by *S. sclerotiorum*. We detected the expression of 74 *PADRE* genes, including 28 *AtPADRE*, 26 *SlPADRE*, and 23 *RcPADRE* (Figure 5 and Supplementary Table S3). Among them, 31 were significantly induced, 11 were significantly down-regulated, and 32 were not differentially expressed. *PADRE* genes highly expressed in healthy leaves were frequent in groups d and h and a subgroup of group f. Group h was very homogenous with all four genes significantly induced upon *S. sclerotiorum* infection, and groups j and f included mostly induced genes, with group d that included a majority of down-regulated genes. To determine whether *PADRE* gene induction differs significantly between phylogenetic groups or between species, we performed ANOVA on expression LFC for the 74 *PADRE* genes. In a one-way ANOVA, the phylogenetic group effect was found highly significant (*p*-value 0.0052) while the species was not significant (*p*-value 0.68). In a two-way ANOVA, the phylogenetic group effect was significant (*p*-value 0.018); the species effect and group × species interaction effect was not significant (*p*-value 0.72 and 0.99, respectively). We conclude that *PADRE* gene expression upon *S. sclerotiorum* inoculation differs between phylogenetic groups in a consistent manner across plant species.

Through a synteny analysis, we identified five pairs (AT1G06980/AT2G30230, AT1G10530/AT1G60010, AT1G210 10/AT1G76600, AT3G03280/AT5G17350, and AT3G10120/AT5G03890) and two quartets AT1G71015/AT2G01340/AT1G66480/AT5G37840, AT2G23690/AT4G37240/AT5G66580/AT3G50800) of *PADRE* genes associated as paralogs in the *A. thaliana* genome. In all instances, groups of paralogs belonged to the same phylogenetic group. The divergence in expression of the *PADRE* genes was limited within most of the paralog groups (Supplementary Figure S1). Only the pair of paralogs AT3G10120-AT5G03890 (group f) showed significant divergence in expression (LFC-5.23 and 3.53, respectively), possibly due to their low basal level of expression. Altogether, our gene expression analysis suggests that responsiveness to pathogens was acquired early in the evolution of Angiosperms by specific groups or subgroups of *PADRE* genes.

### *A. thaliana PADRE* Genes Respond to Multiple Stress and Associate With Plant Defense Ontologies

**FIGURE 6:**
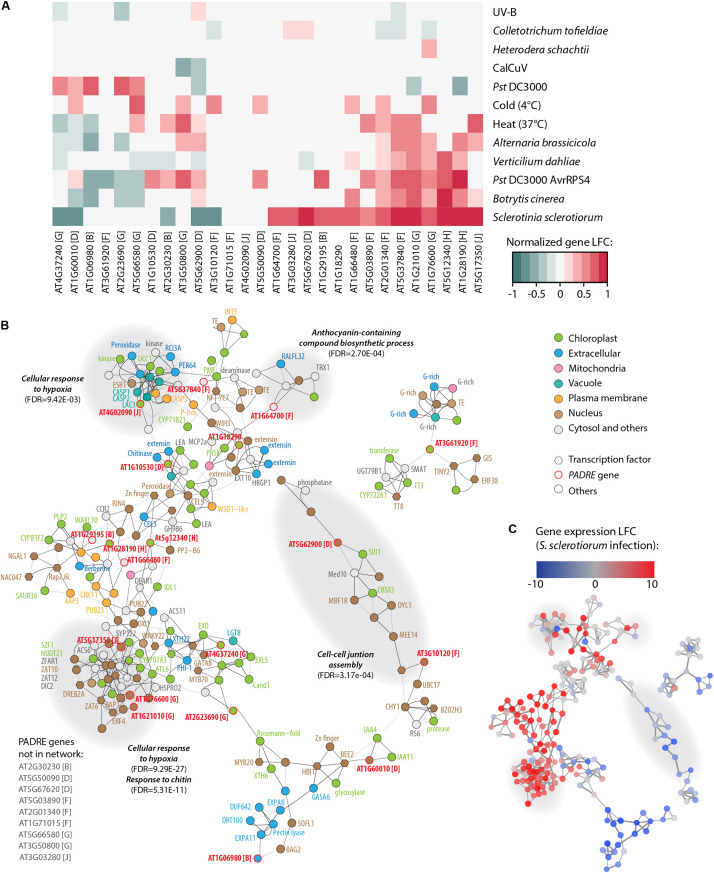
Response to multiple pathogens and co-expression network for *AtPADRE* genes. **(A)** Expression profiles of *A. thaliana PADRE* genes under multiple biotic and abiotic stresses deduced from published RNA sequencing data. The expression levels of genes were normalized using min–max feature scaling to fit within the [–1; 1] range for all experiments. Non-significant LFCs are displayed as 0. The phylogenetic group of *AtPADRE* genes is given between square brackets. The associated raw data is available from GEO accessions GSE132169, GSE70094, GSE72548, GSE56922, GSE88798, GSE112225, GSE85653, GSE83478, GSE104590, GSE116269, GSE66290, and GSE106811. *Pst*, *Pseudomonas syringae* pv. *tomato*. **(B)** Co-expression network for *AtPADRE* genes deduced from an experiment of 14,668 microarrays. Nodes are color-coded according to subcellular localization predicted by WOLF-Psort, shown as hexagons for transcription factors and as circles otherwise. *AtPADRE* genes are outlined and labeled in red. Edge widths are scaled according to a mutual rank score index for co-expression. Gray-shaded areas show a subnetwork identified by network modularity analysis, with associated specific gene ontologies labeled in bold italics. **(C)** The same co-expression network as in **(B)** with nodes color-coded according to LFC of gene expression upon infection by *S. sclerotiorum* determined by RNA sequencing (GEO accession GSE106811). LFC, log_2_ fold change of gene expression.

The finding that some *AtPADRE* genes are responsive to abiotic stresses (Yang et al., 2020) prompted us to investigate their expression under a range of biotic stresses. For this, we analyzed RNA sequencing data available in the Gene Expression Omnibus database (Figure 6A), collecting expression data for *A. thaliana* inoculated by the fungal pathogens *S. sclerotiorum* (Badet et al., 2017), *Botrytis cinerea* (Liu et al., 2015), *Alternaria brassicicola* (Rausch, 2016), and *Verticillium dahliae* (Scholz et al., 2018), the bacterial pathogen *Pseudomonas syringae* pv. *tomato* (*Pst*) DC3000 (Mine et al., 2018) and DC3000 expressing the effector *AvrRps4* (Bhandari et al., 2019), the Cabbage Leaf Curl Virus (CaLCuV) (Zorzatto et al., 2015), and the nematode *Heterodera schachtii* (Shanks et al., 2016). To serve as a reference, we also analyzed RNA sequencing data for plants inoculated with the endophytic fungus *Colletotrichum tofieldiae* (Hacquard et al., 2016) and submitted to heat stress (Albihlal et al., 2018), cold stress (Zuther et al., 2019), and UV-B treatment (Tavridou et al., 2020). The analysis of PADRE gene differential expression revealed a cluster of six *PADRE* genes induced by multiple pathogens: *AT1G28190*, *AT5G12340*, *AT1G76600*, *AT1G21010*, *AT5G37840*, and *AT2G01340* are significantly induced in response to *S. sclerotiorum, B. cinerea, Pst* DC3000 *AvrRPS4*, and *V. dahliae.* Out of these six genes, five are also induced upon infection by *A. brassicicola*, three under heat stress. Three of them are down-regulated in root response to the non-pathogenic fungus *C. tofieldiae*. By contrast, a cluster of five PADRE genes (AT4G37240, AT1G60010, AT1G06980, AT2G23690, and AT5G66580) was down-regulated in response to pathogens and heat stress. The response of *PADRE* genes to heat stress shared more similarities with their response to pathogens than to other abiotic stimuli. *PADRE* genes were not responsive to all signals. Indeed, only *AT2G01340* was differentially expressed in response to the nematode *H. schachtii*, AT3G50800 and AT5G62900 upon infection by the virus CaLCuV, AT1G76600, AT1G21010, AT2G01340, and AT3G10120 in response to *C. tofieldiae*, and AT4G37240, AT3G61920, AT2G23690, and AT1G76600 to UV-B. To test for the relationship between phylogenetic clades and the response of *PADRE* genes to diverse stresses, we performed a two-way ANOVA. We found a significant effect of the phylogenetic group (*p*-value 0.042) and type of stress (*p*-value 4.15 10^–5^) on *PADRE* gene expression. Using a Tukey HSD test, we found that the phylogenetic group effect is due to contrasted expression patterns of genes from groups h and d (*p*-value 0.022). A Tukey HSD test on the stress variable indicated that the stress effect is due to *S. sclerotiorum* infection triggering *PADRE* gene expression significantly different from every other stresses (*p*-value < 0.05), except for *Pst* DC3000 *AvrRPS4* infection and heat stress (*p*-values 0.361 and 0.097, respectively). Therefore, we detected an association (i) between two PADRE phylogenetic groups and responsiveness to multiple stresses and (ii) between the expression of *PADRE* genes and infection by *S. sclerotiorum*. In addition, visual inspection of Figure 6A suggested that *S. sclerotiorum, B. cinerea, Pst* DC3000 AvrRPS4, *V. dahliae, A. brassicicola*, and heat stress induced similar transcriptional responses in *PADRE* genes.

To get insights into genes functioning in the same processes as PADRE genes, we retrieved a co-expression network for *AtPADRE* genes from the ATTED-II database covering 14,668 microarray samples (Obayashi et al., 2018) (Supplementary Datasheet S5 and Figure 6B). The network was composed of 225 nodes and 523 undirected edges, including 19 *AtPADRE* genes. We mapped LFC of gene expression upon *S. sclerotiorum* inoculation obtained from our RNA sequencing analysis onto this network, revealing one major sector including predominantly highly induced genes and another sector including mostly down-regulated genes (Figure 6C). To emphasize biological processes involving *AtPADRE* genes, we performed a modularity analysis based on the network topology (Blondel et al., 2008) to compute subnetworks and test next if every subnetwork corresponded to gene ontology. The modularity analysis identified 12 subnetworks, four of which were significantly associated with a specific biological function (Figures 6B,C). A subnetwork, strongly overexpressed during *S. sclerotiorum* infection (Figure 6C), was involved in the perception of the fungus cell wall and in the response to chitin (FDR = 5.3E-11). Response to hypoxia (FDR = 9.45E-3, FDR = 9.29E-27) was overexpressed during infection by *S. sclerotiorum* whereas genes involved in the cell-cell junction assembly were downregulated (Figure 6C). The subnetwork grouping genes associated with the biosynthesis of anthocyanin (FDR = 2.7E-4), secondary metabolites with antifungal activity (Kumar Sudheeran et al., 2019), appeared overexpressed during infection. To test further the role played by *AtPADRE* genes in the topology of the network, we computed the local centrality (or degree) of every gene (Supplementary Figure S2). Despite the high centrality of the *At5g17350* gene, centralities of AtPADRE genes did not differ significantly from other genes of the network (mean degree AtPadre 4.63, others 4.65, Wilcoxon’s test *p*-value = 0.53).

## Discussion

Plant genomes harbor a remarkably large number of gene families that are not found in other life kingdoms, several of which function in cell signaling (Yamasaki et al., 2013) and defense (Tenhaken et al., 2005; Raffaele et al., 2007; Weidenbach et al., 2016). Through contextual searches, we identified experimental evidence that DUF4228 genes are involved in pathogen and abiotic stress response, cadmium tolerance, disordered region-containing family (Zhang et al., 2016; Yang et al., 2020), and we propose to refer to this family as the PADRE family to reflect these functional and architectural information. Naming domains based on the first functional clues is unlikely to reflect all or the most prominent function of gene families but can foster further research on the function of these genes (Doerks et al., 2000; Habermann, 2004; Tenhaken et al., 2005). Some *PADRE* genes are responsive to environmental stimuli such as wounding and viruses (Akiyama et al., 2005), drought, cold, and salt (Yang et al., 2020), pointing toward yet uncharacterized molecular functions.

### Insights Into the Evolutionary History of the PADRE Domain

We identified 344 high-quality PADRE protein sequences across 13 plant genomes and used this information in a phylogenetic analysis to explore the dynamics of the PADRE domain evolution. Analysis of the extent diversity of PADRE proteins suggests that they originated before the divergence between Bryophyta and Tracheophyta, like an estimated ∼50% of plant-specific domains (Kersting et al., 2012). We classified PADRE proteins into 10 phylogenetic groups, corresponding approximately to subdivisions of the three groups proposed by Yang et al. (2020). In our analysis, the phylogenetic signal was too weak to infer a common ancestor to several groups and combine them with confidence. The BadiRate analysis highlighted a strong radiation of the PADRE domain at the base of the Angiosperms, around ∼350 to 175 million years ago. It should be noted that our dataset does not include sequences from the Pinophyta and Pteridophyta lineages, so that the burst of PADRE diversification may date back to the divergence of these groups or to the Angiosperm most recent common ancestor. The recent duplication of PADRE genes from groups b, d, f, g, and j in *A. thaliana* is well supported by the phylogeny and synteny analysis and consistent with Yang et al. (2020). Recent duplications in these groups are also likely in *M. truncatula*, *S. lycopersicum*, and *A. coerulea*. However, PADRE domain births remained limited or null within the core Eudicot clade, where domain loss seemed predominant. This could indicate selection toward some degree of functional specialization in the PADRE family, favoring the expansion of a few clades to the detriment of the overall domain diversity. Our pangenomic expression analysis supported somewhat consistent patterns of PADRE gene expression upon *S. sclerotiorum* inoculation within phylogenetic groups and across species. This suggests that responsiveness to fungal infection was acquired by PADRE groups f, g, h, and j early in the evolution of core Eudicots. Nevertheless, there was striking contrast in expression within groups f and g, which may indicate some degree of neo- or subfunctionalization.

### A Probable Bipartite Architecture With Structured and Disordered Regions

Sequence analysis pointed toward a bipartite architecture for the PADRE domain, with a combination of structured and intrinsically disordered regions. Intrinsically disordered regions (IDRs) are flexible protein regions lacking a stable 3D fold in solution, which may transition to an ordered state upon binding to natural ligands (Uversky, 2013). Proteins with IDRs are abundant in eukaryotic genomes and are depleted in hydrophobic residues and enriched in polar and charged residues. We found higher amino acid polarity at the C-terminus of PADRE proteins, in agreement with high disorder probability in this region. The peculiar composition and folding properties of IDRs confer specific functional properties (Sun et al., 2013; Uversky, 2013). First, IDRs are generally able to establish protein-protein interactions with multiple partners and are commonly found in hub proteins in eukaryotic networks. One paradigmatic example in plant immunity is RPM1-interacting protein 4 (RIN4) which interacts with multiple plant resistance proteins and bacterial effectors (Sun et al., 2014). In line with this property, PADRE proteins were shown experimentally to interact with calmodulins (Popescu et al., 2007) and response regulators (Dortay et al., 2008). Our co-expression network also suggests a high degree of connectivity for PADRE genes. Screening for protein-protein interactions involving PADRE proteins should prove an insightful avenue for future research. Second, IDRs are highly accessible regions and can therefore undergo complex regulations by post-translational modifications. For instance, Remorins are plant-specific proteins with a role in plant immunity (Raffaele et al., 2009; Bozkurt et al., 2014) containing structured and disordered regions, with their IDRs harboring multiple phosphorylation sites (Marín and Ott, 2012; Marín et al., 2012; Perraki et al., 2018). RIN4 also undergoes multiple post-translational modifications and regulation by proteolysis (Toruño et al., 2019). Similarly, we identified multiple phosphorylated residues in the C-terminal region of PADRE proteins, as well as degradation signals. Third, the ability to undergo a disorder-to-order transition can confer transient functionality to IDRs, such as membrane binding in Remorins (Perraki et al., 2012), cytotoxic activity of *Bordetella* CyaA toxin (O’Brien et al., 2018), and protein complex formation by cAMP response element-binding (CREB) protein (Arai et al., 2015). We could then speculate that every PADRE protein could adopt several functions according to their cellular environment.

### Toward a Functional Understanding of PADRE Family

We report the significant induction of 31 *PADRE* genes upon inoculation by *S. sclerotiorum*, including 14 *AtPADRE*, 7 *RcPADRE*, and 10 *SlPADRE* genes. Radiation of the PADRE family into 10 phylogenetic groups could provide the basis for some degree of functional diversification. In line with hypothesis, Yang et al. (2020) identified 3 *AtPADRE* genes induced upon osmotic stress, 4 upon salt stress, and 5 upon cold stress. Our work revealed an intrinsically disordered region in PADRE proteins, suggesting that PADRE gene function could be context-dependent. This could explain why Yang et al. (2020) found several AtPADRE genes mis-regulated by salt while none were significantly responding to NaCl in the RNA sequencing dataset we analyzed (Suzuki et al., 2016). The identification of multiple subcellular localization signals in PADRE proteins (N-myristoylation, NLS, endocytic vesicles) prevents predictions regarding the site of PADRE action. The use of fluorescent protein reporter fusions in a structure-function analysis will be required to this end. This approach shall be challenging given the presence of targeting signals at both ends of the PADRE domain. We found six *AtPADRE* genes induced upon inoculation by several fungal pathogens with a necrotrophic lifestyle (*S. sclerotiorum, B. cinerea*, and *A. brassicicola*), a bacterial pathogen (*P. syringae* pv. *tomato*), and a hemibiotrophic root-infecting fungus (*V. dahliae*), indicating that pathogens are very potent inducers of *AtPADRE* genes. The PADRE co-expression network included several important players in plant immunity such as the syntaxin SYP122 (Zhang et al., 2007), the C2-domain protein BAP1 (Yang et al., 2006), the patatin-like protein 2 PLP2 (La Camera et al., 2009), members of the RPM1-interacting protein 4 RIN4 (At3g48450), wall-associated kinase-like WAKL10 (At1g79680), and the nematode resistance protein-like HSPRO2 (At2g40000). These findings are consistent with a role for members of the PADRE family in disease resistance.

## Materials and Methods

### Pfam Domain Annotation and Enrichment Analyses

Pfam domains were annotated using hmmscan 3.1b1 with e-value threshold 1E-10 against the Pfam-A 32.0 database. Enrichment of Pfam domains among genes induced after *S. sclerotiorum* infection was analyzed using a two-proportion *Z*-test in R. *Arabidopsis thaliana* gene expression from GEO accession GSE106811 (Badet et al., 2017) was used in this analysis. Briefly, total RNA was extracted from the edge of developed necrotic lesions of leaves from 4-week-old plants inoculated by *S. sclerotiorum* strain 1980, as described in Peyraud et al. (2019). Samples were collected in triplicates from three plants in independent inoculation experiments. RNA sequencing was performed on an Illumina HiSeq 2500 instrument as described in Badet et al. (2017). A composite enrichment score taking into account the significance of the *Z*-test and the enrichment ratio was calculated with the formula *R*_*Z*_(i) ^∗^ *R*_*r*_(i), where *R*_*Z*_(i) is the normalized rank of domain i for the *Z*-test *p*-value and *R*_*r*_(i) is the normalized rank of domain i for the enrichment ratio.

### RNA Sequencing Data Analysis

Raw data for RNA sequencing experiments used in this work is available in the NCBI Gene Expression Omnibus (GEO) database with accession numbers provided in Table 1. All raw datasets were processed separately with DESeq2 to calculate normalized read counts (Basemean) and log_2_ fold change (LFC) of expression and identify genes differentially expressed between control and treated samples. Genes were considered differentially expressed for LFC ≥ 1.5 and adjusted *p*-value ≤ 0.01. In the multiple stress analysis, raw data were used for statistical analysis and LFC values were normalized for the heatmap, as follows: (±)log_2_(1 + |LFC|).

**TABLE 1:** List of experiments used to analyze the response of *A. thaliana PADRE* to multiple biotic and abiotic stresses, with the corresponding GEO accession numbers.

Plants and treatment	Plant age	Time post treatment	GEO accession
*A. thaliana* and *S. lycopersicum* inoculated by *S. sclerotiorum*	4 weeks	48 h	GSE106811
*R. communis* inoculated by *S. sclerotiorum*	4 weeks	50 h	GSE138039
*A. thaliana* inoculated by *Botrytis cinerea*	4 weeks	14 h	GSE66290
*A. thaliana* inoculated by *Alternaria brassicicola*	NA	NA	GSE83478
*A. thaliana* inoculated by *Verticillium dahlia*	14 days	24 h	GSE104590
*A. thaliana* inoculated by *Colletotrichum tofieldiae*	10 days	10 days	GSE70094
*A. thaliana* inoculated by *Pseudomonas syringae* pv. *tomato* DC3000	30 days	24 h	GSE88798
*A. thaliana* inoculated by *Pseudomonas syringae* pv. *tomato* DC3000 AvrRPS4	4 weeks	24 h	GSE116269
*A. thaliana* inoculated by *Heterodera schachtii*	10 days	10 days	GSE72548
*A. thaliana* inoculated by CaLCuV	7 leaf stage	20 days	GSE56922
Heat-treated (37°C) *A. thaliana* Col-0	5 weeks	30 min	GSE85653
Cold-treated (4°C) *A. thaliana* Col-0	28 days	3 days	GSE112225
UV-B-treated *A. thaliana* Col-0	4 days	6 h	GSE132169

*NA, not available.*

### Taxonomic Distribution of PADRE Proteins

We used MAFFT Version 7.407 (Katoh et al., 2002) to align the *Arabidopsis thaliana* DUF4228 protein sequences using default parameters. After manual curation, 24 *A. thaliana* DUF4228 proteins expressed in our RNA sequencing data (GSE106811) were kept for further analysis. This alignment (Supplementary Datasheet S1) was used in a phmmer search on the HMMER webserver^2^ (Potter et al., 2018) against the UniProt References Proteomes in UniProtKB (The Uniprot Consortium, 2019). The search was carried out using parameters -E 1e-10 –domE 1 –incE 1e-10 –incdomE 0.03 –seqdb uniprotrefprot identifying 3467 significant sequence hits. The ‘target length’ and length of the target alignment from the output of the HMM search were used to compare total protein length and DUF4228 domain length (Figure 2A). Genome sizes and total number of genes per genomes were obtained from the Phytozome 12.1 database (Goodstein et al., 2011). Ploidy levels were obtained from the Plant DNA C-values Database on the Kew Royal Botanic Gardens website (Plant DNA C-values Database | Royal Botanic Gardens, Kew) and from the original genome papers. The timetree was generated on http://www.timetree.org/ (Kumar et al., 2017) using species names as input. Polyploidization events described in the literature were collected from Wang et al. (2019) and Xu et al. (2019). DUF4228 proteins in complete plant genomes were identified using hmmsearch against a local instance of the Phytozome 12.1 proteome database, using the same parameters as previously.

### Phylogenetic Analysis of PADRE Proteins

We extracted DUF4228 proteins from *Marchantia polymorpha*, *Physcomitrella patens*, *Sphagnum fallax*, *Selaginella moellendorffii*, *Amborella trichopoda*, *Brachypodium distachyon*, *Setaria italica*, *Aquilegia coerulea*, *Solanum lycopersicum*, *Medicago truncatula*, *Arabidopsis thaliana*, *Ricinus communis*, *Vitis vinifera*, and *Theobroma cacao* from our hmmsearch against Phytozome 12.1. Prior to alignment, we removed sequence Pp3c24_13210 for having <40 amino acids and truncated the 650, 650, and 1650 N-terminal amino acids from Solyc05g013500, Thecc1EG010515, and Medtr8g069400, respectively. A first sequence alignment was performed in ClustalO (Madeira et al., 2019), and 10 sequences were removed for being too divergent, leaving 344 sequences (Supplementary Datasheet S2). These sequences were aligned with ClustalO, and the alignment was manually edited in Jalview to keep positions with no gap in at least 172/344 sequences, yielding a final alignment of 116 amino acids long (Supplementary Datasheet S3). Phylogenetic relationships were determined by a maximum likelihood approach using PhyML (Guindon et al., 2010) with aLRT branch support in phylogeny.fr (Dereeper et al., 2008), with no alignment and no alignment curation steps (Supplementary Datasheet S4), using the LG substitution model (Le and Gascuel, 2008) and a gamma distribution with four categories. The resulting tree had a log-likelihood of −48028.9 and gamma shape parameter 1.720. The tree was rooted on *M. polymorpha* Mapoly0024s0004 and rendered with FigTree^3^ v1.4.3. Phylogenetic groups were defined based on the most ancestral branch with support ≥ 0.9. The rates of birth and death of PADRE domains were calculated using BadiRate 1.35 (Librado et al., 2012) with parameters -bmodel FR -ep CML –family.

### Bioinformatics Analyses of PADRE Sequence Features

Conserved motifs were identified in the 28 *A. thaliana* PADRE proteins using the alignment provided in Supplementary Datasheet S3 and rendered using WebLogo 3 (Crooks et al., 2004). ELMs were identified using the ELM webserver (Gouw et al., 2018) with *A. thaliana* as species and subcellular localization not specified. Phosphorylated peptides were identified with a ‘Basic search’ in ‘Experiment data’ in the PhosPhAt 4.0 database (Durek et al., 2009). Intrinsic disorder probability was calculated using the PrDOS webserver (Ishida and Kinoshita, 2007) with a false-positive rate of 5%. Grantham residue polarity was determined using the ProtScale tool in ExPASy (Gasteiger et al., 2005) with a window size of 9.

### Reconstruction of PADRE Co-expression Network

The co-expression network was built using the NetworkDrawer tool in ATTED-II version 9.2 (Obayashi et al., 2018) using the Ath-m version C7.1 platform including 14,668 microarray samples, with the Coex option “Add many genes” and PPI option “Add a few genes.” The resulting network was rendered in Cytoscape 3.6.1 (Shannon et al., 2003). Gene expression LFC upon *S. sclerotiorum* corresponds to the *A. thaliana* RNA sequencing data from Badet et al. (2017) (GSE106811), with LFC values provided as node attribute table in Cytoscape (Shannon et al., 2003). The modularity of the network was computed by the algorithm proposed by Blondel et al. (2008). Gene ontologies associated with subnetworks were determined using the GO enrichment analysis online tools^4^. Cutoff on FDR was set at 1E-2.

## Data Availability Statement

RNA sequencing read datasets are available from the NCBI Gene Expression Omnibus (GEO) database with accession numbers GSE106811, GSE138039, GSE66290, GSE83478, GSE104590, GSE70094, GSE116269, GSE72548, GSE56922, and GSE72806. The datasets generated by the analyses presented in this study are included in the article Supplementary Material.

## Author Contributions

MD, MK, and SR performed phylogenetic analyses. MD, LG, and SR performed gene expression analyses. AB and SR performed co-expression network analysis. SR conceived and designed the study. All authors contributed to writing the manuscript draft, reviewed the manuscript, and approved the final article.

## Conflict of Interest

The authors declare that the research was conducted in the absence of any commercial or financial relationships that could be construed as a potential conflict of interest.

## Footnotes

1 https://www.i2bc.paris-saclay.fr/maturation/Myristoylome.html

2 https://www.ebi.ac.uk/Tools/hmmer/

3 http://tree.bio.ed.ac.uk/software/figtree/

4 4geneontology.org

## Supplementary Material

FIGURE S1 Genomic distribution and synteny analysis of DUF4228 genes in *A. thaliana*. For each gene, the phylogenetic clade and the level of expression (log_2_ fold change) upon infection by *S. sclerotiorum* is provided. Genes significantly differentially expressed upon infection are indicated with a star (^∗^). Lines in the center of the graph show *PADRE* synteny blocks. The quartet AT2G23690/AT4G37240/AT5G66580/AT3G50800 is reported as two gene pairs in Yang et al. (2020), the quartet AT1G71015/AT2G01340/AT1G66480/AT5G 37840 was not associated with gene duplication by Yang et al. (2020).

FIGURE S2 Modularity of genes included in the AtPADRE co-expression network. Genes with values higher than average are labeled on the graph. Boxplots show first and third quartiles (box), median (thick line) and the most dispersed values within 1.5 times the interquartile range (whiskers).

TABLE S1 PFAM domains enriched in genes induced upon *S. sclerotiorum* inoculation.

TABLE S2 Expression of 24 PADRE genes from *A. thaliana* under a range of biotic and abiotic stress, with mention of their inclusion in the list of genes upregulated upon *S. sclerotiorum* inoculation, down-regulated upon *S. sclerotiorum* inoculation and top 10% most induced genes after infection by *S. sclerotiorum*.

TABLE S3 Expression of 74 *PADRE* genes from *A. thaliana*, *S. lycopersicum* and *R. communis* upon *S. sclerotiorum* inoculation determined by RNA sequencing.

DATASHEET S1 Sequence alignment of 24 *A. thaliana* DUF4228 proteins produced by MAFFT and used for phmmer search, in fasta format.

DATASHEET S2 Full-length sequence of 344 PADRE proteins from 13 Embryophyte species in fasta format.

DATASHEET S3 Sequence alignment of 344 PADRE proteins from 13 Embryophyte species produced by ClustalO and used for phylogenetic analyses, in fasta format.

DATASHEET S4 Maximum likelihood phylogenetic tree of 344 PADRE proteins from 13 Embryophyte species, in in Newick format.

DATASHEET S5 Co-expression gene network shown in Figures 6B,C, in .xgmml format.
