# Supplementary figures and images for: Patterns of Sequence and Expression Diversification Associate Members of the PADRE Gene Family With Response to Fungal Pathogens
Source: Front Genet. 2020 May 29;11:491. doi: 10.3389/fgene.2020.00491 (PMC7272662; doi:10.3389/fgene.2020.00491)

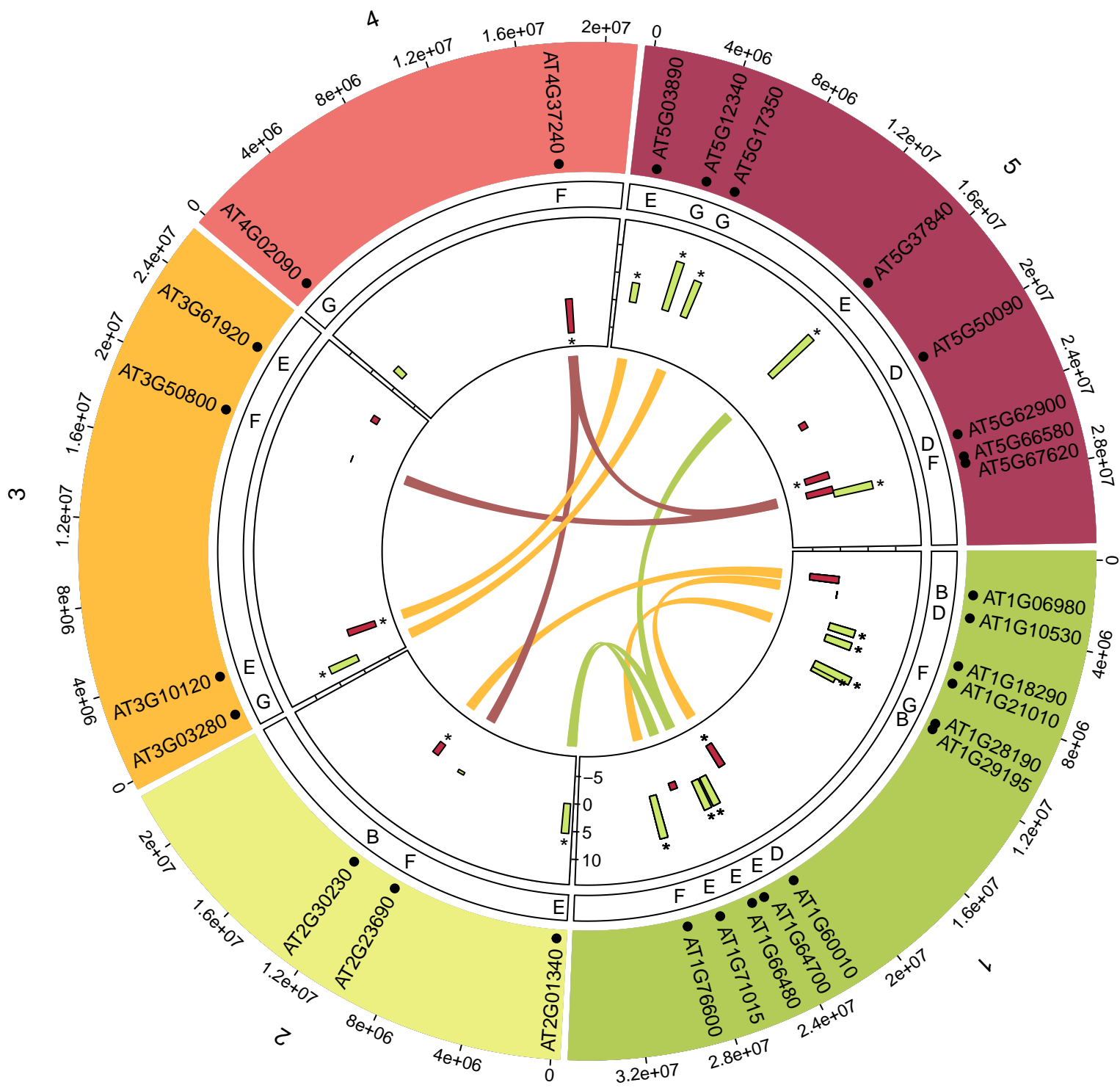

10

5

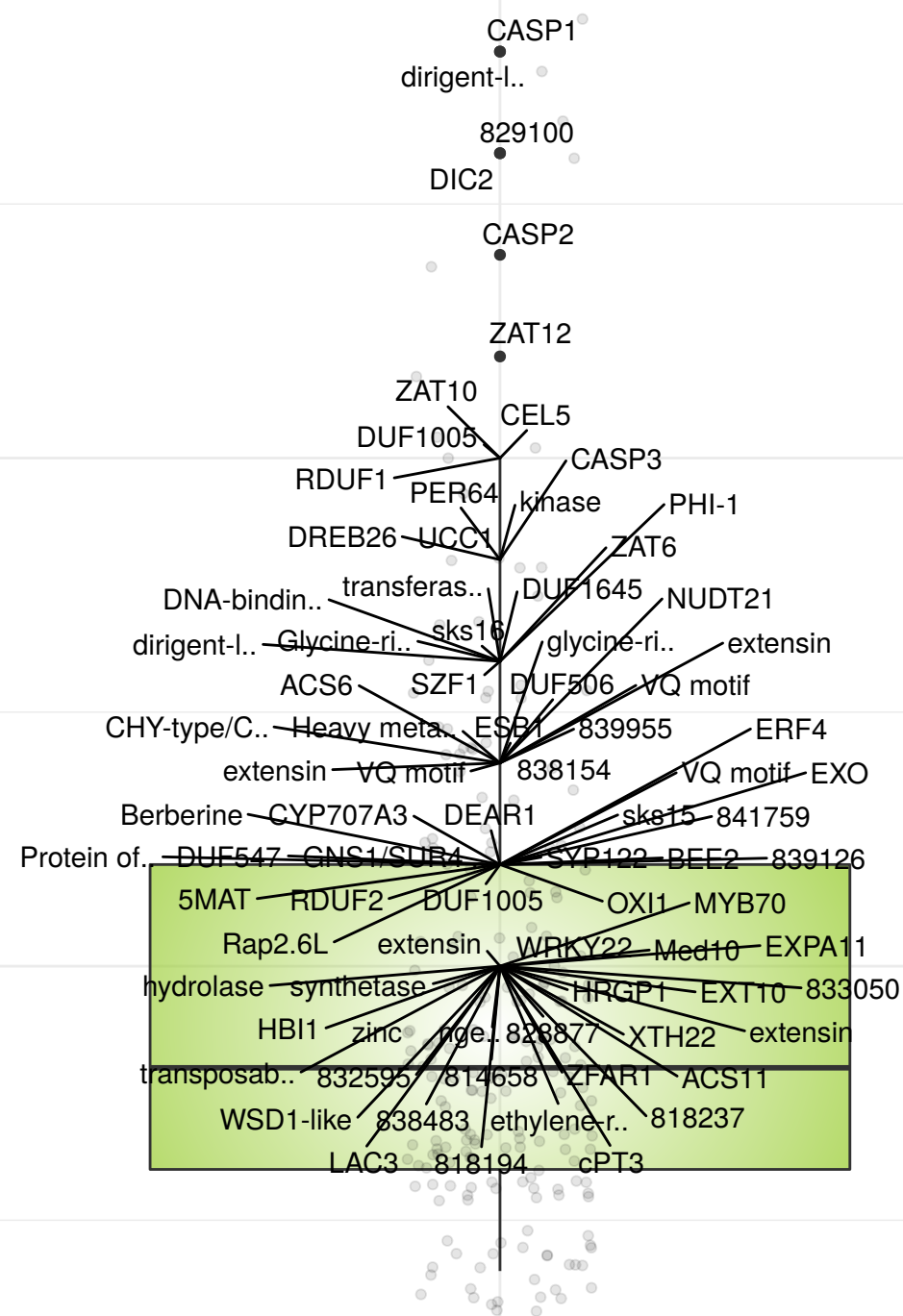

Others

AtPADRE genes

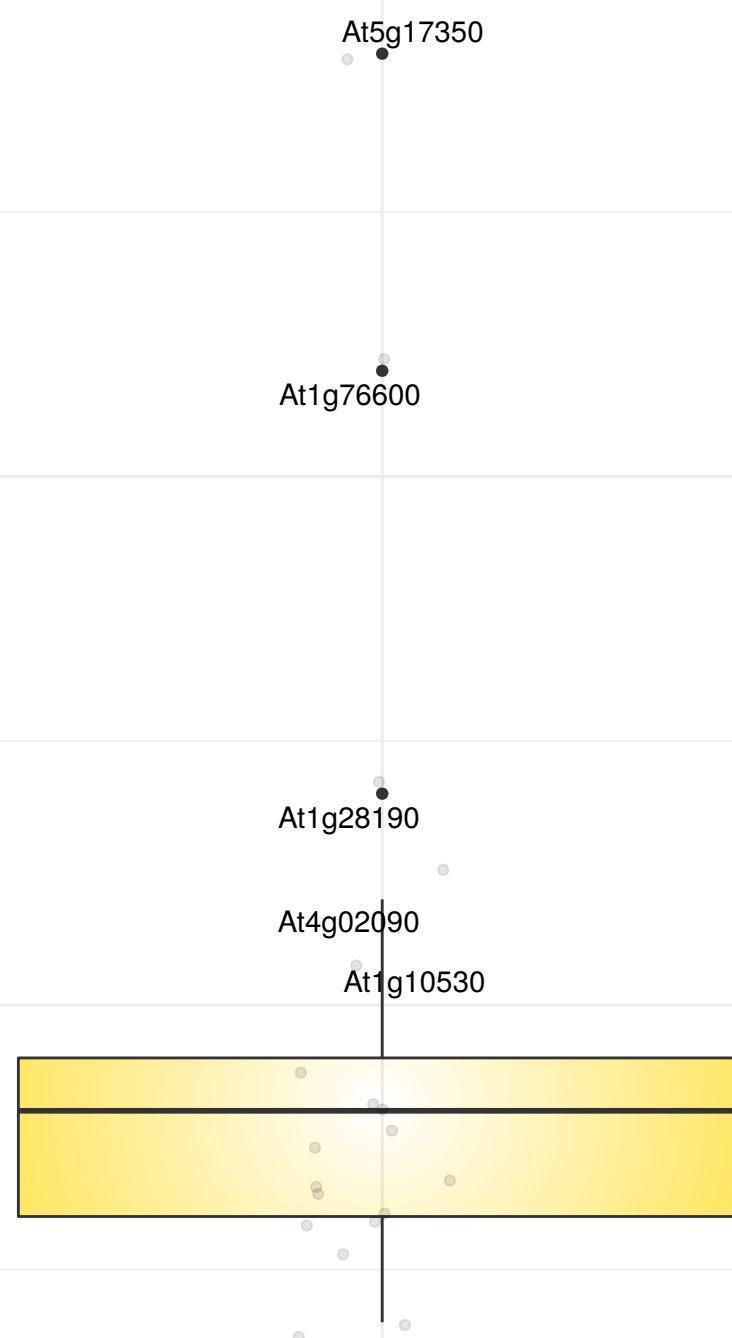

Supplement: FIGURE S1 — Genomic distribution and synteny analysis of DUF4228 genes in A. thaliana. For each gene, the phylogenetic clade and the level of expression (log2 fold change) upon infection by S. sclerotiorum is provided. Genes significantly differentially expressed upon infection are indicated with a star (∗). Lines in the center of the graph show PADRE synteny blocks. The quartet AT2G23690/AT4G37240/AT5G66580/AT3G50800 is reported as two gene pairs in Yang et al. (2020), the quartet AT1G71015/AT2G01340/AT1G66480/AT5G 37840 was not associated with gene duplication by Yang et al. (2020). [file Presentation_1.pdf]
